# Movement Strategies for Countermovement Jumping are Potentially Influenced by Elastic Energy Stored and Released from Tendons

**DOI:** 10.1038/s41598-018-20387-0

**Published:** 2018-02-02

**Authors:** Logan Wade, Glen Lichtwark, Dominic James Farris

**Affiliations:** 1 School of Human Movement and Nutrition Sciences, The University of Queensland, Brisbane, Australia; 2 Sport and Health Sciences, The Univeristy of Exeter, Exeter, UK

## Abstract

The preferred movement strategies that humans choose to produce work for movement are not fully understood. Previous studies have demonstrated an important contribution of elastic energy stored within the Achilles tendon (AT) during jumping. This study aimed to alter energy available for storage in the AT to examine changes in how jumpers distribute work among lower limb joints. Participants (n = 16) performed maximal and sub-maximal jumps under two paradigms, matched for increasing total work output by manipulating jump height or adding body mass. Motion capture and ground reaction force data were combined in an inverse dynamics analysis to compute ankle, knee and hip joint kinetics. Results demonstrated higher peak moments about the ankle joint with added body mass (+26 Nm), likely resulting in additional energy storage in the AT. Work at the ankle joint increased proportionally with added mass, maintaining a constant contribution (~64%) to total work that was not matched with increasing jump height (−14%). This implies greater energy storage and return by the AT with added mass but not with increased height. When total work during jumping is constant but energy stored in tendons is not, humans prioritise the use of stored elastic energy over muscle work.

## Introduction

Navigating the environment requires the coordination of numerous muscles to produce movement. Due to the multiarticular nature of the human body and the synergistic action of muscles spanning body joints, the mechanical work required to produce movement could theoretically be generated by many different muscle coordination patterns. To identify what drives individual movement preferences, we can manipulate factors that we suspect are important by using a movement where such factors are readily manipulated, such as jumping^1–3^. Research in jumping coordination has been primarily focused on maximal jumping, which appears to utilise a single optimal muscular control pattern to reach the maximum jump height^4–7^. This task is complex due to the competing interests of maximizing limb forces and prolonging the time over which these forces are applied to the ground. One key factor in increasing the time in contact with the ground is joint sequencing, where peak muscle forces are generated in a sequence starting at the hip and moving proximo-distally down to the ankle^7–10^. Proximo-distal sequencing also allows for bi-articular muscles to decelerate the proximal joints at the end of their rotation. In this way, bi-articular muscles protect the joints from injury in hyperextension while still allowing proximal muscles to continue activating late into the push-off phase, transferring their power to distal joints along the limb^11–15^. Use of bi-articular muscles in this way maximises jump height by facilitating greater forces being applied to the ground over a longer period of time.

Maximal height jumping has been well studied, however it is also important to understand how humans generate sub-maximal efforts which are required for everyday living. Research into submaximal movements has identified that while an infinite number of activation patterns potentially exist to perform the same movement, a single activation pattern is generally evident for a particular combination of joint moments^7,16,17^. Possible criteria for determining which activation pattern is used include minimizing muscular fatigue^18,19^, minimizing muscular stress^20^ and minimizing metabolic energy consumption^21^. During sub-maximal and maximal jumping, the primary influential factor that dictates which movement strategy is employed is the required jump height. However, in sub-maximal jumping, a highly prioritised secondary influential factor may be introduced in order to better coordinate muscle activation and produce appropriate forces. Vanrenterghem *et al*.^1^ proposed movement effectiveness to minimise energy expenditure as a secondary influential factor for sub-maximal jumping. They concluded that during sub-maximal jumping to increasing jump heights, countermovement depth and rotation of large proximal segments were increased while contribution of work at the ankle was decreased^1^. This was considered a strategy that minimised dissipation of energy at lower jump heights. We consider that it may also be a strategy that reflects the structure-function relationship of leg muscles. This is because the architecture of more proximal limb muscles lends itself to modulating work output through fibre length changes, whereas distal (ankle) muscles rely more on tendon stretch and recoil^22,23^. As such, to increase work output, ankle plantar flexors must first store more energy in their tendons, requiring greater tendon force and ankle moments. Vanrenterghem *et al*.^1^ showed that as total work output increased with jump height, the peak moment about the ankle joint did not change or even slightly decreased. Therefore, from their results we can infer that as work output increases with no corresponding change in peak moment at the ankle, there is likely no change in energy stored in the AT. This may be an additional cause for the decreased work contribution from the ankle with increasing jump height, beyond simply that work at the hip and the knee are increasing. Their original paper was supported by a second study^24^ which used computational simulation, optimising the objective function of jump height (primary influential factor) in combination with minimizing muscle work (secondary influential factor). The simulation demonstrated a reduction in muscular work with decreasing jump height, due to the improved relative use of the series elastic element (SEE) of the plantar flexors performing a higher percentage of total work^24^. It is therefore conceivable that work is redistributed to favour proximal joints for higher jumps as ankle plantar flexors are unable to increase work contribution via storage and return of energy in the AT. However, it is worth considering how humans might alter their strategy for producing work if more energy were available for storage in the AT.

The importance of series elastic tissues for jumping has been well documented^25,26^. Muscles are limited in how much force they can produce due to constraints imposed by the force-velocity and force-length relationships. Such constraints are not imposed on the SEE^27^. The AT therefore enables high shortening velocities and power outputs of the plantar flexor muscle-tendon units at the end of push off^28^, allowing the muscle fibres to contract and perform work more slowly over their optimal range while a large amount of energy can be released rapidly by the SEE. Human experimental^29,30^ and simulation data^28^ suggests that elastic energy stored in the triceps surae tendon enhances work output by allowing muscles to contract closer to their optimal shortening velocity. This is possible because unlike stiffer tendons such as those located at the hip, the AT is compliant, enabling high amounts of elastic energy storage that can be returned later in the movement at an explosive speed. Previous research found that 35–40% of the work from the ankle was delivered by the AT^25,26^, therefore the energy stored in the AT has great potential to influence jumping coordination. Farris *et al*.^30^ proposed that the majority of the energy stored within the SEE of the triceps-surae was stored against the resistance of body weight, prior to or during the jumping movement. Given the aforementioned benefits of using stored elastic energy, it seems logical that when additional elastic energy is available we would prefer to use it over work from contractile tissues.

Therefore, the aim of this study was to examine if changing the energy available to be stored in the SEE of the triceps-surae would influence the ratio of joint work contributed at the ankle to the overall movement. To examine this we manipulated the mechanical work requirements of jumping in two different ways: (1) Body Mass Paradigm (BMP) - Altering body mass (for a constant jump height) to manipulate the work required for jumping; (2) Jump Height Paradigm (JHP) - Altering jump height to provide a comparable manipulation of total work. Based on Farris *et al*.^30^ it was expected that increasing work via adding mass would increase ankle moments and energy potentially stored in the AT. Based on Vanrenterghem *et al*.^1^ it was expected that increasing work via greater jump heights would require increased work contributions of the proximal joints, while ankle moments and elastic energy stored within the SEE of the triceps surae remain constant. Therefore we hypothesised that increasing total work via increased jump height in the JHP would result in participants adopting a movement strategy that favoured contributions at proximal joints, whereas equivalent increases in total work via increased body mass would result in a movement strategy that relied more on work at the ankle joint.

## Results

Both paradigms achieved their intended purpose with BMP showing no change in jump height and JHP increasing from a height of 0.291 m up to 0.476 m (Table 1). This increase in jump height was primarily a product of distance travelled in the air as take-off height stayed relatively constant across all conditions (Table 1). A Two way ANOVA showed a significant main effect of increasing work on the countermovement depth (P < 0.001) for both paradigms (Table 1), however there was not a significant main effect of paradigm (P = 0.578) or interaction affect (P = 0.530). Thus there was no statistical difference in the countermovement depth between the JHP and the BMP as work output increased and direct comparisons can be made.

**Table 1:** Group mean ± SD of jump height metrics of the two experimental paradigms.

	Body Mass Paradigm				Jump Height Paradigm			
	100%	120%	140%	160% (Maximal)	Jump Height 1	Jump Height 2	Jump Height 3	Maximal
Total Jump Height (m)	0.294 ± 0.038	0.289 ± 0.038	0.290 ± 0.040	0.291 ± 0.039	0.294 ± 0.038	0.344 ± 0.040	0.411 ± 0.051	0.476 ± 0.042
Flight Height (m)	0.189 ± 0.037	0.186 ± 0.035	0.179 ± 0.036	0.167 ± 0.040	0.189 ± 0.037	0.240 ± 0.036	0.299 ± 0.046	0.360 ± 0.045
Toe Off Height (m)	0.105 ± 0.020	0.104 ± 0.023	0.112 ± 0.022	0.124 ± 0.027	0.105 ± 0.020	0.105 ± 0.024	0.112 ± 0.024	0.116 ± 0.023
Countermovement Depth (m)	−0.180 ± 0.055	−0.217 ± 0.059	−0.244 ± 0.063	−0.285 ± 0.063	−0.180 ± 0.055	−0.225 ± 0.060	−0.251 ± 0.060	−0.314 ± 0.053

### Peak joint moments

**Figure 1:**
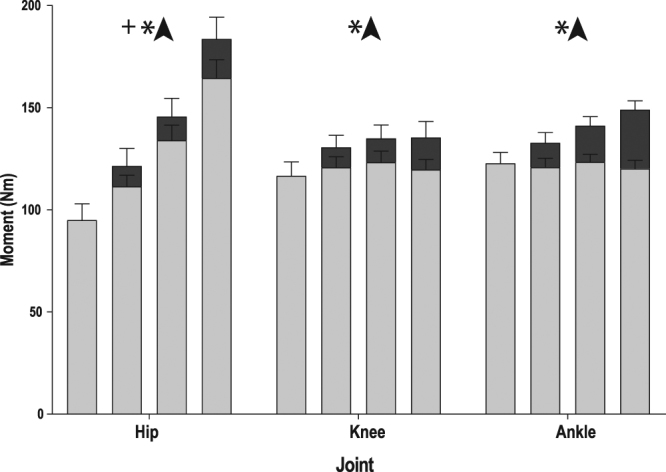
Peak (±SEM) joint moments of the hip, knee and ankle with increasing work (from left to right) under each jumping paradigm (JHP: light shading, BMP: dark shading). Significant main effect of increasing work on peak joint moment is represented by + for JHP and * for BMP.

**Figure 2:**
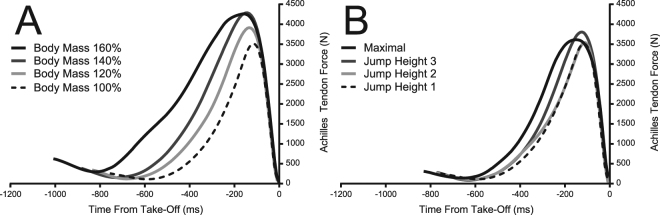
Mean time series graphs of the estimated Achilles tendon force in the BMP (**A**) and the JHP (**B**). Time zero occurs at the point the toes leave the ground. Time series data begins at the start of the countermovement phase.

Peak joint moments are reported for the right leg only (Fig. 1). The peak moment about the ankle increased in the BMP, resulting in a significant main effect of added mass (P < 0.001). Peak moment started at 123 Nm in the shared condition and increased to 149 Nm in the 160% body mass condition. There was no significant main effect of jump height on peak ankle moment (P = 0.459) in the JHP. Peak knee moment increased in the BMP from 117 Nm in the shared condition up to 135 Nm in the maximal 160% body mass condition (P < 0.001). Peak knee moment did not change significantly with increasing jump height (P = 0.311). Peak hip joint moments increased under both paradigms with a higher increase in the BMP. The shared condition started at 95 Nm and increased to 183 Nm at 160% body mass in the BMP (P = <0.001) and 164 Nm at maximal jump height in the JHP (P < 0.001). Estimations of AT force (Fig. 2) with increasing jump height showed no difference in peak force (P = 0.045, α = 0.0167), starting at 3661 N in the jump height 1 condition and finishing at 3901 N at the maximal jump height condition. Increasing mass in the BMP did result in a significant main effect of peak AT force (P < 0.001), increasing from 3661 N in the shared condition up to 4499 N in the 160% body mass condition.

### Joint kinematics

**Figure 3:**
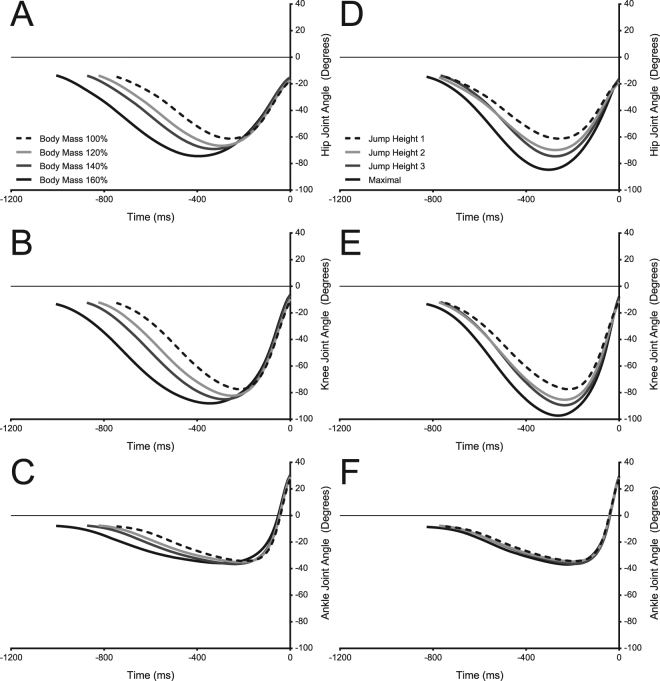
Mean time series graphs of joint angles of the hip (**A** & **D**), knee (**B** & **E**) and ankle (**C** & **F**) for the BMP (**A**,**B**, and **C**) and the JHP (**D**,**E** and **F**). Time zero occurs at the point the toes leave the ground. Time series data begins at the start of the countermovement phase. Hip and knee extension (**A**,**B**,**D**,**E**) is in the positive direction. Ankle plantarflexion (**C**,**F**) is in the positive direction.

As total work increased, the joint range of motion for all joints (hip, knee and ankle) significantly increased, regardless of paradigm (P < 0.001). Subtracting the peak JHP (Fig. 3A,B and C) angles from the peak BMP (Fig. 3D,E and F) angles for each joint it can be seen that the JHP range of motion increased by 10.4 degrees more in the hip (P < 0.001), increased by 9.4 degrees at the knee (P < 0.001) and did not change significantly at the ankle (P = 0.0326, α = 0.0167).

### Joint work

**Figure 4:**
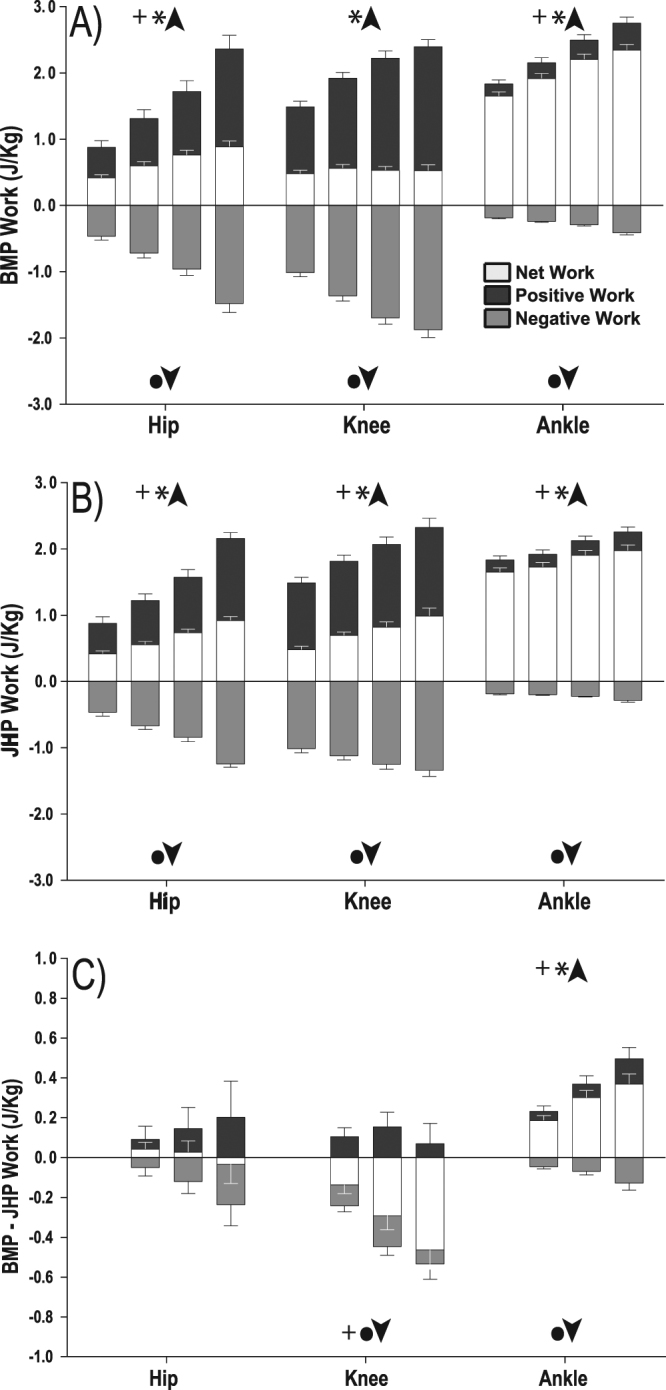
Total (±SEM) net, positive and negative work per kilogram (normalised to unweighted body mass) of the hip, knee and ankle for increasing BMP paradigm (**A**), JHP paradigm (**B**) and the difference between corresponding conditions in each paradigm (**C**). Significant main effect of increasing work by either increasing body mass or increasing jump height are represented by + (net work), *(positive work), ● (negative work). ↑ Indicates a significant increase, ↓ indicates a significant decrease.

Increases in total work (Fig. 4A,B) resulted in all joints demonstrating an increase in net work across both paradigms (P < 0.001, Fig. 4) with the exception of the knee in the BMP where there was no change in net joint work (P = 0.573, Fig. 4A). Positive and negative joint work increased significantly in all joints under both paradigms (P < 0.001, Fig. 4A,B). Figure 4C shows the difference in work done at each joint between BMP and JHP with each increment of total work. Therefore, each bar in Fig. 4C represents the subtraction of the joint work value for a given jump height in the JHP, from the joint work value from the matched added mass condition in the BMP (Fig. 4A). As such, any values in Fig. 4C that are not zero indicate that joint work varied differently at that condition in the BMP and JHP. There was no significant change in the difference between the BMP and JHP in net (P = 0.581), positive (P = 0.370) and negative (P = 0.053) work (Fig. 4C) by the hip joint as total work increased. There was also no significant change in difference between the JHP and BMP in positive work (P = 0.326) at the knee joint as total work increased. However the difference between the JHP and BMP for net work (P < 0.001) and negative work (P < 0.001) at the knee joint became more negative with increasing total work (Fig. 4C). Finally, the difference between JHP and BMP in net (P < 0.001) and positive (P < 0.001) work at the ankle joint was significantly greater as total work increased, paired with a smaller but significant increase in the difference in negative work at the ankle joint between the two paradigms (P = 0.006).

### Joint work contributions

**Figure 5:**
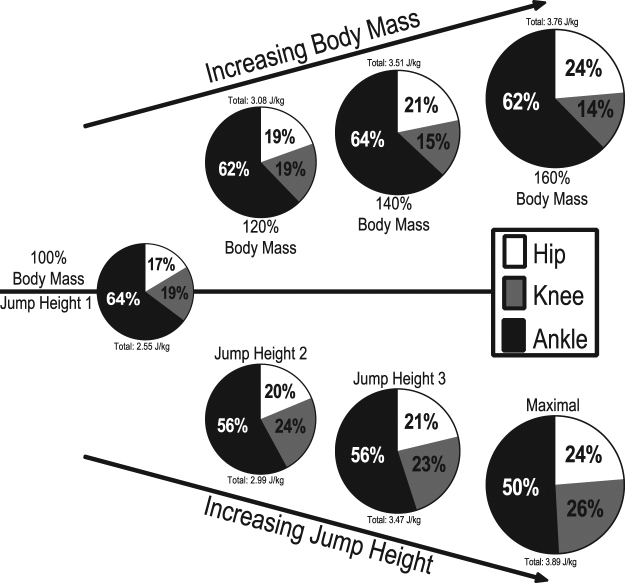
Pie graphs depicting the joint work contributions of the ankle, knee and hip during each sub-condition. The total area of each pie graph is indicative of the total amount of work produced by all three joints, relative to the other pie graphs.

In this section, work contributions refers to the percent contribution of an individual joint to the total net work of the ankle, knee and hip joints combined. Increasing work in the JHP resulted in a 14% (P < 0.001) reduction in work contribution by the ankle joint, a 7% (P < 0.010) increase in work contribution by the knee joint and a 7% (P < 0.001) increase in work contribution by the hip joint between the shared condition and maximal jump height (Fig. 5). Additional body mass resulted in no significant change in work contribution by the ankle joint (P = 0.140) while the work contribution of the knee joint decreased by 5%, although this was not statistically significant (P = 0.054). Work contribution of the hip joint increased 7% (P < 0.001) between the shared condition and 160% body mass condition. Thus, we can see that at 160% body mass, the ankle contributes 62% of the work to the entire movement, whereas at maximal jump height with 100% body mass only 50% of the work is contributed by the ankle, even though the total work output of the entire movement is the same (Fig. 5).

## Discussion

It was proposed that adding mass to a participant during jumping would result in additional storage of energy in the AT, inferred from observing an increase in peak moment about the ankle joint and an increase in estimated AT force. There was a significant main effect of additional body mass in the BMP on the peak moment about the ankle. Peak moment about the ankle increased by 26 Nm between the shared condition and 160% maximal body mass condition. With no increase in body mass in the JHP there was no change in the peak moment about the ankle joint. Estimated peak force at the AT also showed no significant difference in the JHP while there was a significant increase in the AT peak force in the BMP. With an increase in peak force at the AT, the tendon was placed under a higher tension and thus had to store additional energy which could be used later in the movement. Based on these results, we infer that there was additional energy stored within the AT as a consequence of added mass applied to the body and that this additional energy storage did not occur with increasing jump height.

With additional energy stored in the AT due to added mass, we expected to see an increase in the positive work at the ankle joint in the BMP compared to the JHP. Our results confirmed this hypothesis, as the work (net, positive and negative) at the ankle joint increased significantly in the BMP compared to the JHP (Fig. 4C & Fig. 4). We believe that a greater proportion of the total work was generated at the ankle joint in the BMP than in the JHP, due to the preferential use of energy stored in the series elastic tissues of the plantar flexors. Assuming this energy can be returned, this likely resulted in the observed additional positive work at the ankle joint with increased body mass. There was no difference between the net, positive and negative work at the hip joint between both paradigms and therefore the hip does not seem to be affected by additional mass during countermovement jumping (Fig. 4C). There was no significant difference in positive work at the knee joint between paradigms (Fig. 4C). Alternatively, net work of the knee was higher in the JHP than in BMP, this was due to greater negative work in the BMP (Fig. 4C). This large negative work was produced during the countermovement phase of the BMP jump, when the knee extensors are decelerating the body. This increase in negative work has also been found in drop jump experimentation, where the negative work at the knee joint increases due to resisting the larger inertial forces imparted on the body as a product of dropping from greater heights^31^. This increase in negative work was not matched by a similar increase in positive work and thus it appears that the knee is absorbing and dissipating this energy as opposed to storing and returning it.

There was a significant increase in the peak moment about the knee in the BMP which was not evident in the JHP, increasing by 19 Nm from the 100% added mass condition to the maximal condition. Previous studies have shown that the SEE of the knee stretches under load, resulting in decoupled length changes of the MTU and fascicles, indicating the ability to store elastic energy at this joint^32,33^. However, evidence for increased energy return at the knee was not apparent in this study, as there was no difference in positive work output in both paradigms. One possible reason for this absence is that energy return from the elastic structures is delivered late in the movement, when knee velocity is required to be reduced in order to protect the knee capsule from rupture at the end of range of motion^11^. Therefore it is possible that instead of absorbing and dissipating the additional negative energy stored in the BMP, the knee could return this energy during this late deceleration phase, where kinetic energy from the knee is being transferred down to the ankle via the biarticular gastrocnemius^13,34^.

Total work increased comparably between paradigms and while the work at the ankle joint in the BMP increased proportionally, it did not under the JHP. The ankle joint maintained a constant net work contribution across all added mass’s (64–62%), while under the JHP the net work contribution at the ankle decreased by 14% as jump height increased (64% down to 50%; Fig. 5). We believe that the constant work contribution at the ankle joint under the BMP was a product of additional energy stored within the AT, due to resisting additional mass. Thus participants altered their joint contribution strategy, presumably to enable the use of all the energy stored within the tendon. In contrast, the JHP resulted in no increase in peak moment about the ankle or estimated AT force and therefore no likely change in elastic energy stored at the AT. This conclusion is supported by Vanrenterghem *et al*. (2008) who found no change in elastic energy storage with increasing jump height in their simulation paper^24^. In order to increase the total work output of the body with increasing jump height alone, the work contribution of the hip and knee joints are increased, allowing for the required high power outputs to be generated by the large proximal muscles attached to relatively stiffer tendons^35^. This matches our results under the JHP as we also saw an increase in net work contribution at the hip and knee joint. We also found that the hip and the knee peak angle increased by a further 10 degrees in the maximal condition of the JHP compared to the maximal condition of the BMP. This was paired with relatively no change in the peak angle of the ankle. Thus, the body moves away from an ankle centred movement strategy, towards a knee and hip centred movement strategy. Vanrenterghem *et al*. (2004) found a similar result with an increase in jump height alone, identifying a shift in joint contribution strategy that was dominated by the proximal joints.

That humans prioritise energy efficient movement strategies is not a new theory. It has long been established that the transition from walking to running occurs at the speed where walking becomes more energetically costly than running, due to high shortening velocities of the muscle and use of increased energy stored at the AT made possible by running above certain speeds^36,37^. This was also shown in hopping where the body’s natural hopping frequency and leg stiffness optimised the use of energy stored within the tendon and minimised the cost of creating muscular force, even on a range of compliant surfaces^38,39^. Furthermore, research examining hopping with passive exoskeletons has demonstrated that muscles will reduce their activation and force output, allowing the exoskeletal device to perform the missing portion of the work to match the original total work output^40,41^. The findings described here add to this literature, further expanding our knowledge of how humans prioritise the choice of movement patterns during sub-maximal movements. In this study, additional energy is stored in the AT as a result of resisting higher inertial loads. The participant then has the option of performing movement with the same joint coordination they normally would and dissipating the energy stored in the tendon, or using this energy and changing their movement strategy. Under these circumstances the participant appears to change their movement strategy and prioritises not wasting energy that is already stored. The compliance of the tendons at each individual joint appears to have a great impact on the potential for increasing work output as tendons are not limited by the same length or velocity constraints that affect muscle fibres. Thus, with additional mass and possibly more energy stored in the tendon, the muscles may be able to perform a larger portion of the work at slower speeds, over a more optimal range of motion^42^. In order to truly understand how additional body mass affects the storage of energy within the lower limb tendons, more direct measures of muscle activation and shortening are required.

Due to the highly specific nature of controlling jump height, the study required a large volume of jumping from participants. All possible measures were taken to make sure that participants had adequate rest and were not performing too many jumps. This paper has made no direct measures of muscle activation patterns, tendon length changes, or alternatively the length changes of muscle fascicles and therefore inferences about energy storage were made based on joint moments and modelled estimates of tendon force. The AT exhibits elastic properties and we are therefore confident in stating that increases in tendon force necessitate increased energy storage. However, we were unable from the present data to quantify increases in energy storage. Possible other reasons for joint contribution redistribution such as altering of the COM due to added mass, change in joint speeds or increase in positive ankle work due to biarticular energy transfer from proximal muscles cannot be rejected based on the data in this paper. However our observations are consistent with our theory.

Additional body mass added via a weight vest increased the peak moment about the ankle joint and therefore potentially increased elastic energy stored in the AT. In the JHP, the ankle work output did not increase sufficiently to maintain the same work contribution percentage with increasing total work output, thus the hip and knee contribution increased. Alternatively, in the BMP the ankle joint maintains a constant work contribution percentage, likely due to increased energy stored within the SEE. This difference in work contribution between paradigms demonstrates the humans’ preference for using all available energy within the elastic structures over mechanical energy that must be produced by muscles. Furthermore, there was no significant difference in the positive work of the hip and knee joints between the two paradigms. Further research is required to examine the storage and production of energy at a muscular level in order to confirm this theory.

## Methods

### Protocol

**Figure 6:**
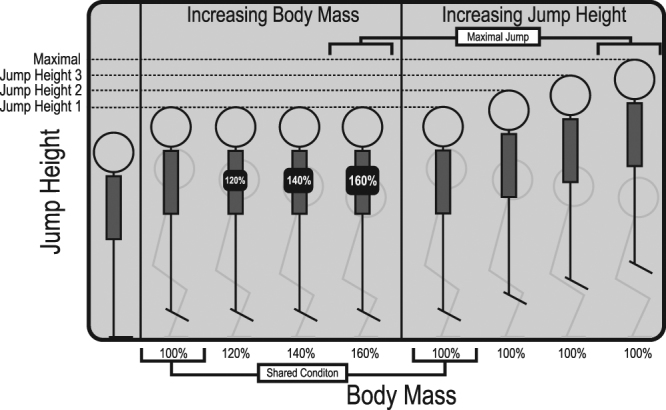
Jumping conditions split into two paradigms of either increasing body mass at a constant jump height or increasing jump height at a constant body mass.

Written informed consent was obtained from male participants (N = 16, height = 179 ± 4.6 cm, mass = 74.2 ± 5.8 kg, age = 23.7 ± 3.3) to participate in this study. All participants self-reported that they were healthy and had not been injured within the last 12 months. This study aimed to assess changes in joint coordination during jumping in the general population and therefore participants were recreationally active but were not trained jumpers. Ethical approval was granted from the institutional ethics review committee at The University of Queensland and all methods were performed in accordance with the relevant guidelines and regulations. Participants performed vertical countermovement jumps with preferred countermovement depth over a range of experimental conditions designed to manipulate the total mechanical work required for each jump. Work was manipulated either by adding mass to the participant, varying jump height or both. Target jump heights were determined based on the maximal jump height achieved with each of the 4 added masses within this study (Fig. 6). Thus, jump height 1 was determined by the maximal jump achieved with 160% body mass, jump height 2 was determined by the maximal jump achieved with 140% body mass, jump height 3 was determined by the maximal jump height achieved with 120% body mass and the maximal jump height was determined by the maximal jump achieved with 100% body mass (no mass added). Participants were first randomly assigned one of the four mass conditions. Once the maximal jump height at that mass was determined the participant completed jumps with all remaining lower masses to the same height in a random order. Thus for jump height 1, participants first jumped maximally with 160% body mass and then matched this jump height with 100%, 120% and 140% body mass in a random order (Fig. 6). Randomizing the conditions first by jump height and then by added mass increased the reliability of the participants to accurately match the required jump height, reducing the total number of jumps performed. This jumping protocol enabled comparisons to be made between one experimental paradigm (BMP) and one control paradigm (JHP) (Fig. 6). Each increment in these paradigms represented an equivalent increase in the total mechanical work output, starting from jump height 1 with no added mass, which will hence forth be referred to as the shared condition, before diverging and increasing up to the maximal conditions for each paradigm, matching for total work output with each increment.

Added mass was achieved by placing up to two weight vests on the participant. Weight vests could be loaded with up to thirty, individual 1 kg blocks, for a combined maximum of 60 kg of added mass. Visual feedback for each jump height was provided via a light box which was used in conjunction with verbal feedback of the exact jump height, calculated live from ground reaction force data. The light box consisted of a double slit and a row of LED lights located at the back of the box, ensuring that the only time the lights could be seen was when the participants’ eyes were directly horizontal to the LED lights.

Prior to data collection, participants were familiarised with the jumping protocol and mechanisms for controlling body mass and jump height. This included placing an empty weight vest on their chest, instructing them on how to perform the countermovement jump without arms and moderate their own jump height using the light box. They were given as much practice at sub-maximal jumping with the light box as they liked in order to make sure they were comfortable with the setup and confident in their ability to target specific jump heights.

During experimentation participants performed at least three jumps at each condition, however sub-maximal jumps were only deemed successful if they were calculated to be within 1 cm of the target height. After each sub-maximal jump the researcher informed the participant if the jump was within the targeted height and if not, how far outside the target their jump was. All trials were recorded but only successful trials were used in data analysis. Each condition was completed when three successful jumps were recorded or the participants had performed ten jumps at that condition. Under this protocol all conditions recorded at least one successful jump per participant. Sub-maximal conditions were allocated at least 30 seconds between each jump to allow for adequate rest. Maximal jumping conditions did not require a target height and therefore were only performed three times with 90 seconds of rest. Participants were still informed of their jump height after each maximal jump as a motivational tool.

### Experimental data collection

An eight camera, three-dimensional (3D) optoelectronic camera system (Oqus, Qualisys, AB, Sweden) was used to collect motion capture data. Reflective markers were placed on the body for static and dynamic measures. Markers were placed on both legs at the distal phalanx of the first toe, metacarpal phalangeal joints 1 and 5, calcaneus, medial and lateral malleolus of the ankle, medial and lateral joint centre of rotation of the knee, left and right anterior superior iliac spine (ASIS) and posterior superior iliac spine (PSIS), coccyx, vertebrae C7, suprasternal notch of the manubrium and on the acromion process of the left and right shoulders. Clusters of 4 markers on rigid plates were placed on the lateral side of the shank and thigh of each leg midway between joints using Velcro straps. Finally, an empty weight vest was placed on the subject, completing the setup and allowing for their unweighted mass to be calculated. Participants assumed a quiet standing posture with hands locked onto the side of the weight vest while static collection was performed. Static collection trials were used to scale a generic musculoskeletal model during data analysis (detailed below). All experimental trials included this quiet standing posture for 2 seconds before jumping. During all trials, marker position data was sampled at 200 Hz.

Ground reaction force data was recorded at 2000Hz using two force plates located within an instrumented treadmill (Instrumented Tandem Treadmill, AMTI, MA, USA) with one foot placed on each force plate. Jump height and countermovement depths were calculated live using a custom LabVIEW script (National Instruments Corporation, Austin, USA). LabVIEW and Qualisys recording software read the data from AMTI force plate amplifiers (AMTI Gen 5, AMTI, MA, USA). Live jump height calculations were recorded and processed in LabVIEW using force data sampled at 1000 Hz.

### Data analysis

Using the Qualisys software (QTM, Qualisys, AB, Sweden), markers were labelled for each trial and kinetic and kinematic data was exported to OpenSim. A generic OpenSim model, previously described by Hamner, Seth & Delp^43,44^, was modified to remove the upper arms, forearms and hands with their masses added to the head and trunk segment and the cervical joint locked. Segment lengths of the foot, shank, thigh, pelvis and trunk were measured using the distance between individual marker pairs. Marker pairs were as follows: foot - calcaneus to distal phalanx of the first toe, shank - lateral malleolus to lateral knee joint line, thigh - lateral knee joint line to ASIS marker for each leg, pelvis - ASIS to PSIS, left ASIS and PSIS to right ASIS and PSIS, trunk - PSIS to shoulder markers and left shoulder marker to right shoulder marker. Scaling factors were calculated using these distances divided by the distance between these markers on the generic model. Each segment’s length and mass were then scaled according to their respective ratio with the generic model, keeping distribution of masses the same as in the generic model. Once scaling of the model was performed in OpenSim, inverse kinematics analyses were completed using a weighted least-squares fit of the model markers to the experimental markers during jumping trials, allowing for joint angles to be calculated at each time point^45^. Inverse kinematic results for joint angles were then combined with ground reaction force data in an inverse dynamics analysis to calculate joint moments of the ankle, knee and hip. Moment arm of the AT was calculated at each instant throughout the jumping movement as the shortest distance between ankle joint centre of the participant specific model and the line of action of the model GAS and SOL muscles. GAS and SOL moment arms were then averaged to determine a single AT moment arm. AT force was calculated from dividing the ankle moment by the AT moment arm at each time point^46^. Filtering of inverse kinematic marker data and inverse dynamic force data was completed using a 25 Hz second order two-way Butterworth filter. The central difference technique was used to differentiate joint angles and resulting joint velocities were multiplied by joint moments to calculate joint powers. Joint level time-series data was trimmed from the start of the countermovement phase through to take-off. Left and right joint powers were then summed and integrated to calculate joint work. Net work was the integral of all power values from the start of the countermovement until take-off. Positive work was the integral of all positive power values from the start of the countermovement until take-off. Negative work was the integral of all the negative power values from the start of the countermovement until take-off. Individual joint work contributions to total work were calculated by dividing individual net joint work by the summed net joint work of the hip, knee and ankle. Due to the unique protocol allowing total work output to be matched as we increased either jump height or added mass, it is possible to compare directly between the net, positive and negative work at ankle joint in the JHP and the BMP. Thus if we were to subtract the net work at the ankle joint in the JHP from the net work in the BMP, we would see the difference in net work produced by the ankle joint in the BMP compared to the JHP. This may give a clearer indication about how the ankle is changing in net, positive and negative joint work between the two paradigms. Any reference to difference in joint work between paradigms later in this paper is referring to this comparison.

Data from conditions performed at sub-maximal intensities were averaged based on the number of trials that were collected for a specific participant. Where possible three trials were averaged, however if the participant did not achieve three successful trials in that condition, the data was averaged across two trials or in some cases only one trial was used. Participants performed three jumps for each maximal trial, however only the trial that achieved the maximal jump height was used in data analysis. Final results depicts data averaged from all 16 participants.

Jump height was calculated using an integration method from ground reaction force data which was imported into Matlab (Mathworks, MA, USA). Vertical ground reaction forces of each force plate were summed and then body weight (calculated from the vertical ground reaction force during the 2 seconds of quiet standing prior to each jump) was subtracted from the entire time series to calculate the net vertical force. To ensure minimal integration drift error, data was first cropped to the start of the countermovement and the instant of take-off. Start of the countermovement was defined as the point at which the net vertical force dropped to −20 N. Take-off was defined as the point at which the vertical force dropped below 20 N. This ensured that integration was only performed over the shortest time possible (maximum of 1 second). Net vertical force was divided by the participants’ body mass (including any added mass) to find acceleration. Acceleration was then integrated from the start of the countermovement to the time of take-off to identify velocity at take-off. Vertical flight distance was calculated using:

$${\rm{s}}\,=\frac{{v}^{2}-u^{2}}{2a}$$

where final velocity (v) equals 0, initial velocity (u) equals the velocity of the centre of mass at take-off and acceleration (a) = −9.8 m/s². Jump height was defined as the displacement of the centre of mass between standing and the apex of the jump. Due to ankle rotation, the centre of mass was higher at take-off than during standing. To calculate the displacement from standing to take-off, velocity was integrated to find the displacement of the centre of mass at take-off. Flight distance and take-off displacement were then summed to calculate the total jump height. Countermovement depth is the distance between the centre of mass at standing and the bottom of the countermovement.

### Statistical analysis

Two way ANOVA’s were used to examine main and interaction effects of jump height or added mass on net work contribution, net work, positive work, negative work, peak moments, peak joint angle of the hip, knee and ankle joints. Two way ANOVA’s for joint analysis that reported a significant main or interaction effect (alpha 0.05) then had post hoc analysis performed on individual joints. Post hoc analysis was performed using a one way ANOVA with a Bonferroni adjusted alpha level (alpha = 0.0167) on individual joints to determine a main effect of either increasing body mass or increasing jump height. All statistical tests were conducted in GraphPad Prism software (GraphPad Software Inc, California, USA).

### Data availability

Using data from the right leg, moments and angles of the hip, knee and ankle joint for each participant in each condition have been uploaded as supplementary data. Data is normalised to 101 points ending at the instant of take-off. Time stamps for each data point have also been uploaded should they be required.

## Electronic supplementary material

Dataset 1

## References

[1] Vanrenterghem J, Lees A, Lenoir M, Aerts P, De Clercq D (2004). Performing the vertical jump: Movement adaptations for submaximal jumping. Human Movement Science.

[2] van Soest AJ, Roebroek M, Bobbert MF, Huijing P (1985). & van Ingen Schenau, G. J. A comparison of one-legged and two-legged countermovement jumps. Medicine and science in sports and exercise.

[3] Moir GL, Gollie JM, Davis SE, Guers JJ, Witmer CA (2012). The effects of load on system and lower-body joint kinetics during jump squats. Sports Biomechanics.

[4] Asmussen, E. & Sørensen, N. THE ≪WIND-UP≫ MOVEMENT IN ATHLETICS. *Le travail humain*, 147–155 (1971).

[5] Bobbert MF, Casius LJR (2005). Is the effect of a countermovement on jump height due to active state development?. Medicine and Science in Sports and Exercise.

[6] Abbott BC, Aubert XM (1952). The force exerted by active striated muscle during and after change of length. The Journal of Physiology.

[7] Bobbert MF, Schenau VI (1988). G. J. Coordination in vertical jumping. Journal of Biomechanics.

[8] Gregoire L, Veeger HE, Huijing PA, van Ingen Schenau GJ (1984). Role of Mono- and Biarticular Muscles in Explosive Movements. Int J Sports Med.

[9] Pandy MG, Zajac FE (1991). Optimal muscular coordination strategies for jumping. Journal of Biomechanics.

[10] Bobbert MF, van Soest AJ (2001). Why Do People Jump the Way They Do?. Exercise and Sport Sciences Reviews.

[11] van Ingen Schenau, G., Bobbert, M. & van Soest, A. in Multiple Muscle Systems (eds JackM Winters & SavioL- Y. Woo) Ch. 41, 639–652 (Springer New York, 1990).

[12] van Soest AJ, Schwab AL, Bobbert MF, van Ingen Schenau GJ (1993). The influence of the biarticularity of the gastrocnemius muscle on vertical-jumping achievement. Journal of Biomechanics.

[13] Jacobs R, Bobbert MF, van Ingen Schenau GJ (1996). Mechanical output from individual muscles during explosive leg extensions: The role of biarticular muscles. Journal of Biomechanics.

[14] Bobbert MF, Gerritsen K, Litjens M, van Soest AJ (1996). Why is countermovement jump height greater than squat jump height. Med Sci Sports Exerc.

[15] Ettema GJ, Huijing PA, de Haan A (1992). The potentiating effect of prestretch on the contractile performance of rat gastrocnemius medialis muscle during subsequent shortening and isometric contractions. J Exp Biol.

[16] van Bolhuis BM, Gielen CCAM (1998). & van Ingen Schenau, G. J. Activation patterns of mono- and bi-articular arm muscles as a function of force and movement direction of the wrist in humans. The Journal of Physiology.

[17] Prilutsky BI, Zatsiorsky VM (2002). Optimization-Based Models of Muscle Coordination. Exercise and sport sciences reviews.

[18] Dul J, Johnson GE, Shiavi R, Townsend MA (1984). Muscular synergism—II. A minimum-fatigue criterion for load sharing between synergistic muscles. Journal of Biomechanics.

[19] Crowninshield RD, Brand RA (1981). A physiologically based criterion of muscle force prediction in locomotion. Journal of Biomechanics.

[20] An K, Kwak B, Chao E, Morrey B (1984). Determination of muscle and joint forces: a new technique to solve the indeterminate problem. Journal of biomechanical engineering.

[21] Alexander RM (2000). Energy-Minimizing Choices of Muscles and Patterns of Movement. Motor Control.

[22] Biewener AA (2016). Locomotion as an emergent property of muscle contractile dynamics. J Exp Biol.

[23] Ward S, Eng C, Smallwood L, Lieber R (2009). Are Current Measurements of Lower Extremity Muscle Architecture Accurate?. Clin Orthop Relat Res.

[24] Vanrenterghem J, Bobbert MF, Casius LJR, De Clercq D (2008). Is energy expenditure taken into account in human sub-maximal jumping? - A simulation study. Journal of Electromyography and Kinesiology.

[25] Anderson FC, Pandy MG (1993). Storage and utilization of elastic strain energy during jumping. Journal of Biomechanics.

[26] Bobbert MF, Huijing PA, van Ingen Schenau GJ (1986). An estimation of power output and work done by the human triceps surae musle-tendon complex in jumping. Journal of Biomechanics.

[27] Kurokawa S, Fukunaga T, Fukashiro S (2001). Behavior of fascicles and tendinous structures of human gastrocnemius during vertical jumping. Journal of Applied Physiology.

[28] Bobbert MF (2001). Dependence of human squat jump performance on the series elastic compliance of the triceps surae: A simulation study. J Exp Biol.

[29] Kurokawa S, Fukunaga T, Nagano A, Fukashiro S (2003). Interaction between fascicles and tendinous structures during counter movement jumping investigated *in vivo*. Journal of Applied Physiology.

[30] Farris, D. J., Lichtwark, G. A., Brown, N. A. T. & Cresswell, A. G. The role of human ankle plantar flexor muscle-tendon interaction & architecture in maximal vertical jumping examined *in vivo*. *J Exp Biol*, 10.1242/jeb.126854 (2015). 10.1242/jeb.126854 26685172

[31] Bobbert. Drop jumping. II. The influence of dropping height on the biomechanics of drop jumping. *Medicine and science in sports and exercise* **19**, 339 (1987). 3657482

[32] Austin N, Nilwik R, Herzog W (2010). *In vivo* operational fascicle lengths of vastus lateralis during sub-maximal and maximal cycling. Journal of Biomechanics.

[33] Ichinose Y, Kawakami Y, Ito M, Kanehisa H, Fukunaga T (2000). *In vivo* estimation of contraction velocity of human vastus lateralis muscle during “isokinetic” action. Journal of Applied Physiology.

[34] Van Ingen Schenau GJ, Bobbert MF, Rozendal RH (1987). The unique action of bi-articular muscles in complex movements. Journal of Anatomy.

[35] Yamaguchi, G., Sawa, A., Moran, D., Fessler, M. & Winters, J. A survey of human musculotendon actuator parameters. *Multiple muscle systems: Biomechanics and movement organization*, 717–773 (1990).

[36] Sawicki GS, Lewis CL, Ferris DP (2009). It pays to have a spring in your step. Exerc Sport Sci Rev.

[37] Alexander RM (1991). Energy-saving mechanisms in walking and running. J Exp Biol.

[38] Farley CT, Blickhan R, Saito J, Taylor CR (1991). Hopping frequency in humans: a test of how springs set stride frequency in bouncing gaits. J Appl Physiol.

[39] Ferris DP, Farley CT (1997). Interaction of leg stiffness and surface stiffness during human hopping. Journal of Applied Physiology.

[40] Farris DJ, Robertson BD, Sawicki GS (2013). Elastic ankle exoskeletons reduce soleus muscle force but not work in human hopping. Journal of Applied Physiology.

[41] Grabowski AM, Herr HM (2009). Leg exoskeleton reduces the metabolic cost of human hopping. Journal of Applied Physiology.

[42] Roberts TJ, Azizi E (2011). Flexible mechanisms: the diverse roles of biological springs in vertebrate movement. The Journal of Experimental Biology.

[43] Hamner SR, Seth A, Delp SL (2010). Muscle contributions to propulsion and support during running. Journal of Biomechanics.

[44] Dorn TW, Schache AG, Pandy MG (2012). Muscular strategy shift in human running: dependence of running speed on hip and ankle muscle performance. The Journal of Experimental Biology.

[45] Delp SL (2007). OpenSim: Open-Source Software to Create and Analyze Dynamic Simulations of Movement. IEEE Transactions on Biomedical Engineering.

[46] Lichtwark GA, Wilson AM (2005). *In vivo* mechanical properties of the human Achilles tendon during one-legged hopping. J Exp Biol.

